# Case report: Complete response to pembrolizumab in a liver metastatic colon adenocarcinoma patient with a novel likely pathogenic germline MSH2 mutation

**DOI:** 10.3389/fimmu.2022.1064488

**Published:** 2022-11-28

**Authors:** Yanjie Xu, Qin Li, Jiemin Zhao, Xuefeng Ni, Ping Li, Wenwei Hu

**Affiliations:** ^1^ Department of Oncology, The Third Affiliated Hospital of Soochow University, Changzhou, China; ^2^ Geneplus-Beijing, Beijing, China

**Keywords:** Lynch syndrome, colorectal cancer, MSH2, germline mutation, PD-1 inhibitors

## Abstract

Lynch syndrome (LS) is a genetic disorder mainly caused by germline mutations in mismatched repair (MMR) genes (*MSH2*, *MLH1*, *MSH6*, and *PMS2*) or deletions of the epithelial cell adhesion molecule gene (*EPCAM*). A 43-year-old Chinese male patient underwent radical surgery and was pathologically confirmed to have stage IIIB colon adenocarcinoma. After four cycles of standard adjuvant chemotherapy, the tumor reoccurred *in situ* with intestinal obstruction. The patient received secondary colectomy. Immunohistochemistry analysis revealed a loss of MSH2 protein expression in the surgical specimen. Noticing that the patient’s mother and grandfather all were diagnosed with LS-related cancers, we collected the patient’s and his mother’s peripheral blood for genetic testing, and the result showed a six-base deletion of *MSH2*. Thus, we concluded that our patient had LS. Subsequently, the patient accepted pembrolizumab as the first-line systemic therapy after liver metastases. He achieved clinical complete response (cCR) within 2 months and remained progression-free for more than 2 years. The case report showed that *MSH2* mutation (c.489_494deTGGGTA) is a likely pathogenic mutation, and immunotherapy (pembrolizumab) is effective for this patient.

## Introduction

Lynch syndrome (LS), previously also known as hereditary non-polyposis colorectal cancer (HNPCC), accounts for 2%–4% of all colorectal cancer (CRC) cases. LS is an autosomal dominant disorder and the most common genetic syndrome in colorectal cancers, which results in a significantly higher risk of colorectal cancer and other LS-related tumors, including cancer of the endometrium, ovary, stomach, small bowel, pancreas, prostate, and so on (1, 2).

The main cause of LS is a germline mutation in one of the four mismatch repair (MMR) genes (*MLH1*, *MSH2*, *MSH6*, and *PMS2*), and a large fragment deletion of the epithelial cell adhesion molecule (*EPCAM*) gene which leads to methylation of the promoter of *MSH2* and results in gene silencing of *MSH2* can also cause LS (3). Mutations of *MSH2* and *MLH1* are the most frequently reported, accounting for 40% and 50%, respectively (4). There are currently >8,900 *MSH2* mutation entries in the InSiGHT database (https://www.insight-group.org/) and >5,100 items in the ClinVar database (https://www.ncbi.nlm.nih.gov/clinvar/). Truncating mutations are the main causes of the loss of MMR function, comprising about 68% of all *MSH2* mutations (5). The deficiency of the MMR protein is called dMMR.

According to NCCN guidelines (6), all patients with a personal history of CRC are recommended to undergo universal MMR or MSI testing, and patients with MSI-H/dMMR are recommended pembrolizumab or nivolumab, alone or in combination with ipilimumab, as the first-line treatment. The recommendation was based on the data from the Keynote-177 study, in which 11% of the MSI-H/dMMR CRC patients achieved complete responses and 33% achieved partial responses in the pembrolizumab arm, and the percentage was 4% and 29% in the chemotherapy arm, respectively. It also demonstrated a statistically significant improvement in progression-free survival (7).

Here, we reported a patient with LS clinically diagnosed according to the Amsterdam II criteria. Genetic analysis identified a novel germline mutation of *MSH2* (c.489_494delTGGTA), which was reported as a variant of uncertain significance (VUS). The patient was treated with pembrolizumab and acquired clinical complete response (cCR).

## Case description

**Figure 1:**
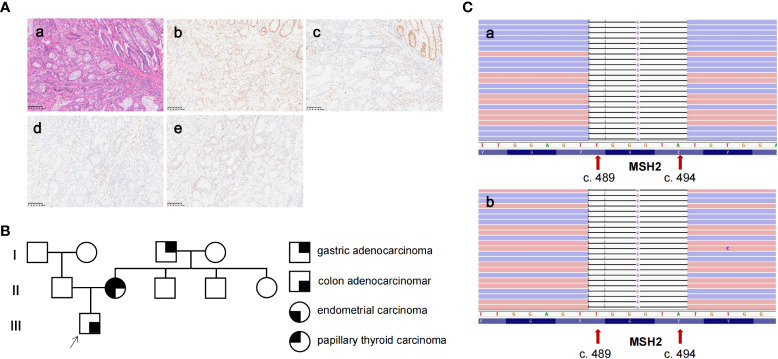
The pathological analysis, genetic testing, and pedigree of the patient. **(A)** (a) H&E staining showing a poorly differentiated colonic adenocarcinoma of the primary tissue (×200). (b–e) IHC staining showed *MLH1* expression, loss of expression of *MSH2* and *MSH6*, and partly the expression of PMS2 in primary tissue (×200). **(B)** The pedigree of the patient. The patient was diagnosed with colon adenocarcinoma at 43 years of age. The patient’s mother was diagnosed with endometrial adenocarcinoma, gastric adenocarcinoma, and papillary thyroid carcinoma at 40, 61, and 63 years of age, respectively. His grandfather was diagnosed with gastric adenocarcinoma at 40 years of age. **(C)** The genetic testing result of the patient (a) and his mother (b) by next-generation sequencing revealed a six-base deletion of *MSH2* (NM_000251.2: exon 3: c.489_494delTGGGTA).

**Figure 2:**
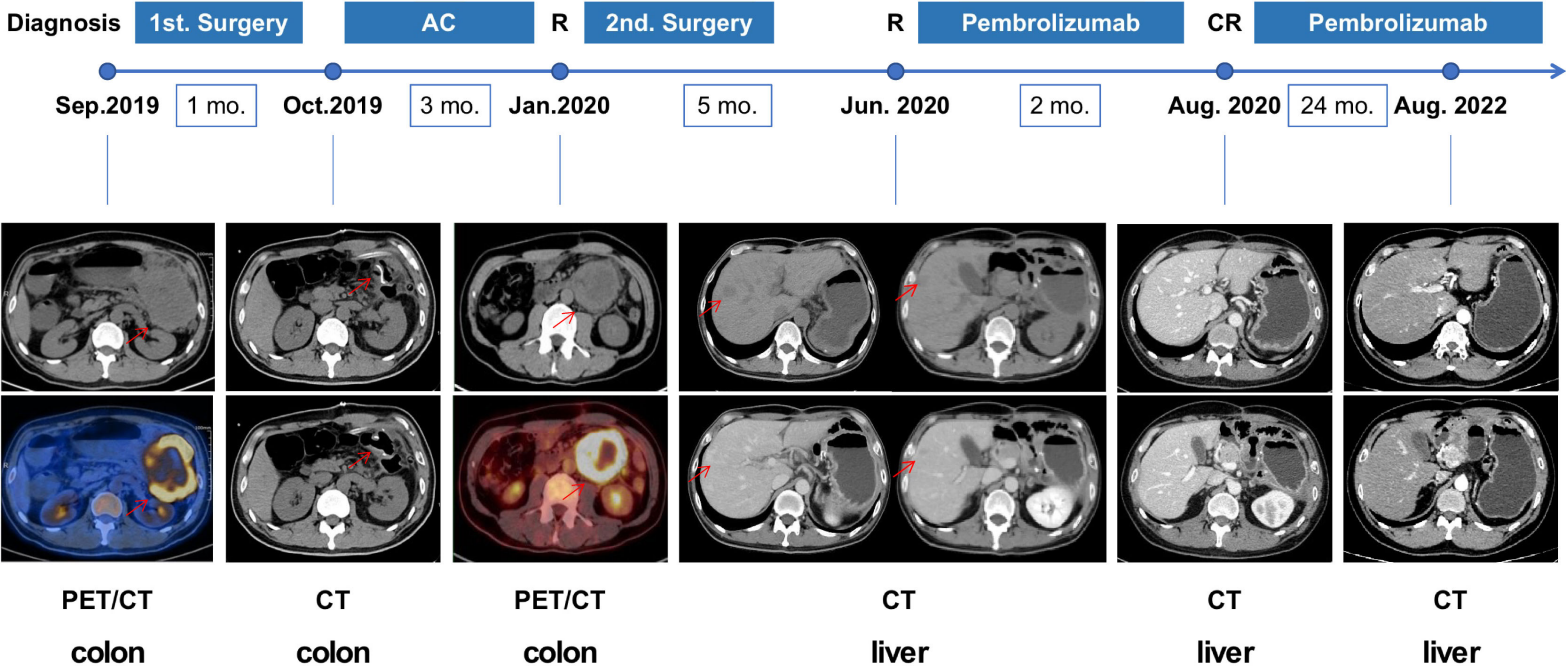
The course depicting the treatment process and radiological evaluation from 2019 to 2022. The patient has received pembrolizumab since June 2020, achieving clinical CR after 2 months and has remained in CR until now. AC, adjuvant chemotherapy; CR, complete response; R, recurrence; CT, computed tomography; PET/CT, positron emission tomography/computed tomography.

A 43-year-old Chinese male patient with left-sided colon adenocarcinoma underwent radical surgery in September 2019. The patient declared no past history of any chronic disease, no smoking history, and no alcohol or drug use. The pathological stage was pT4N1aM0, IIIB according to the 8th edition of the American Joint Committee on Cancer (AJCC) staging system. Immunohistochemical analysis of the MMR protein expression showed loss of MSH2 and MSH6 protein expression in the surgical specimen (**Figure 1A**). After four cycles of standard adjuvant chemotherapy (oxaliplatin and capecitabine), the tumor reoccurred *in situ* quickly, presenting with intestinal obstruction in January 2020. Then, the patient received colectomy again. The secondary surgical specimen was checked for microsatellite instability (MSI) status, and the result showed MSI-H. During regular postoperative examination, CT scan revealed multiple liver metastases in June 2020. When the patient was admitted to our hospital, his vital signs were in the normal ranges, and no abnormal finding was referred during physical examinations. Our laboratory results found no anemia, no white blood cell elevation, and no thrombocyte abnormality. Moreover, no renal, hepatic, thyroid, adrenal, or pituitary function abnormality was reported. The longest diameters of the liver metastases measured by the CT scan were 1.9 and 1.3 cm, respectively. Subsequently, the patient has received programmed death 1 (PD-1) inhibitor monotherapy (pembrolizumab 200 mg q3w) in our hospital since June 2020. He achieved cCR (no signs of tumor in the CT scan) in August 2020. Blood tests (every month) and CT scans (every 3 months) were performed routinely, and no side effect was observed during the immunotherapy. The therapy was well tolerated, and the patient has remained progression-free until now (**Figure 2**).

In consideration of the patient’s diagnosis, rapid recurrence, and MSI status, we investigated his family history according to clinical screening of LS. The patient’s mother was diagnosed with endometrial adenocarcinoma, gastric adenocarcinoma, and papillary thyroid carcinoma at the age of 40, 61, and 63, respectively. His grandfather was diagnosed with gastric adenocarcinoma at 40 years of age (**Figure 1B**). The patient’s family history matches the Amsterdam II criteria, and he was clinically diagnosed to have LS. Consequently, we collected peripheral blood both from the proband and his mother to undergo genetic testing for the *MSH2*, *MLH1*, *MSH6*, and *PMS2* genes. Genetic testing indicated a germline *MSH2* deletion in the reading frame, c.489_494delTGGGTA, resulting in the deletion of two amino acids from 164 to 165 (**Figure 1C**).

This novel mutation has been neither reported in previous studies nor recorded in the InSiGHT, ClinVar, the 1000 Genomes Project (1000G/1KGP), and the Exome Aggregation Consortium (ExAC) databases. Consequently, according to the 2015 American College of Medical Genetics and Genomics and the Association for Molecular Pathology (ACMG-AMP) guidelines for variant classification (8), MSH2 c.489_494delTGGGTA can be classified as VUS.

## Discussion

The clinical diagnosis of Lynch syndrome has depended on the Amsterdam I criteria developed in 1991 (9). Because its sensitivity and specificity were 60% and 70%, respectively (10–12), the Amsterdam II criteria were revised, developed in 1997, and included extracolonic malignancies with the aim to increase sensitivity (13). Otherwise, the revised Bethesda guidelines were used to determine which colorectal cancer patients should undergo MSI gene analysis and MMR immunohistochemistry (14). For patients satisfied with the Amsterdam criteria, direct next-generation sequencing (NGS) should be considered. For patients that meet only the Bethesda guidelines, MSI and/or MMR proteins by immunohistochemistry were first to be proceeded, followed by sequencing if positive. In this case, the proband was diagnosed with colon adenocarcinoma at 43 years of age, and his mother and grandfather were diagnosed with LS-related cancers before 50 years of age. According to the Amsterdam criteria II, this particular family was a typical LS pedigree with at least three successive generations diagnosed with pathologically proven HNPCC and other LS-related cancer, and the genetic testing of MMR genes confirmed the presence of LS.

Among all the mutations related to LS, studies have consistently found that germline mutations in *MLH1* and *MSH2* account for a sizeable majority (60%–80%) of cases, with a relative minority of cases reporting germline mutations in *MSH6* and *PMS2* and germline mutations in *EPCAM*, which is particularly rare (2). In this case, we identified a novel germline mutation in *MSH2* (c.489_494delTGGGTA). The immunohistochemical result of the proband revealed the absence of MSH2 protein expression, and the MSI status of the proband was highly unstable, which both supported the loss of function of *MSH2*. The same genetic mutation also occurred in his mother, who suffered from three kinds of other LS-related tumors. Based on the two strong lines of evidence, the novel *MSH2* germline mutation (c.489_494delTGGGTA) may be “pathogenic” or at least “likely pathogenic.”

According to a series of clinical trials, it was confirmed that dMMR/MSI-H tumors are sensitive to immune checkpoint inhibitors. Furthermore, in the latest study, it seemed that sporadic dMMR/MSI-H CRC patients benefited less from ICIs than patients with LS. The pathological CR (pCR) rate was 58% and 78%, respectively (6). However, the underlying mechanism of different responses to immunotherapy between these two types of dMMR/MSI-H tumors remained unclear. In our case, the patient with LS received PD-1 inhibitor monotherapy (pembrolizumab) after liver metastases. He achieved cCR within 2 months and has remained progression-free until now, benefiting greatly from immunotherapy. This suggests that MMR testing in combination with gene sequencing is helpful to identify LS patients and to choose the precise treatment. The patient is now receiving pembrolizumab treatment every 3 weeks as maintenance therapy. The duration of maintenance therapy remained controversial. Most studies suggested that patients benefiting from PD-1 inhibitors should receive at least 2 years of monotherapy (15, 16). In CRC, the data of maintenance therapy were limited and worthy of further exploration.

## Conclusion

The present case report indicates a novel germline mutation of *MSH2* (c.489_494delTGGGTA). The mutation of *MSH2* is considered a likely pathogenic mutation. The patient with LS acquired great benefits from immunotherapy.

## Data availability statement

The original contributions presented in the study are included in the article/supplementary material. Further inquiries can be directed to the corresponding author.

## Ethics statement

The studies involving human participants were reviewed and approved by Institutional Review Board of The Third Affiliated Hospital of Soochow University. The patients/participants provided their written informed consent to participate in this study. Written informed consent was obtained from the individual(s) for the publication of any potentially identifiable images or data included in this article.

## Author contributions

WH conceptualized and designed the study. YX and QL wrote the manuscript and drafted the figures. JZ, XN and PL were responsible for the data collection, data interpretation, and manuscript preparation. All authors took part in the critical review of the manuscript. All authors contributed to the article and approved the submitted version.

## Acknowledgments

We owe thanks to the patient and his family members for agreeing to the publication of this case report. This work was supported by Changzhou “14th Five-Year Plan” High-level Health Personnel Training Project (No. KY20221357), Changzhou Young Talent Project (No. CZQM2020033), and Changzhou Science and Technology Program (No. CJ20210106).

## Conflict of interest

Authors QL and PL were employed by company Geneplus-Beijing.

The remaining authors declare that the research was conducted in the absence of any commercial or financial relationships that could be construed as a potential conflict of interest.

## Publisher’s note

All claims expressed in this article are solely those of the authors and do not necessarily represent those of their affiliated organizations, or those of the publisher, the editors and the reviewers. Any product that may be evaluated in this article, or claim that may be made by its manufacturer, is not guaranteed or endorsed by the publisher.
